# Hyperspectral imaging for seed quality and safety inspection: a review

**DOI:** 10.1186/s13007-019-0476-y

**Published:** 2019-08-08

**Authors:** Lei Feng, Susu Zhu, Fei Liu, Yong He, Yidan Bao, Chu Zhang

**Affiliations:** 1 College of Biosystems Engineering and Food Science, Zhejiang University, Hangzhou, 310058 China; 2 Key Laboratory of Spectroscopy Sensing, Ministry of Agriculture and Rural Affairs, Hangzhou, 310058 China

**Keywords:** Hyperspectral imaging, Seed quality, Seed safety, Multivariate analysis

## Abstract

Hyperspectral imaging has attracted great attention as a non-destructive and fast method for seed quality and safety assessment in recent years. The capability of this technique for classification and grading, viability and vigor detection, damage (defect and fungus) detection, cleanness detection and seed composition determination is illustrated by presentation of applications in quality and safety determination of seed in this review. The summary of hyperspectral imaging technology for seed quality and safety inspection for each category is also presented, including the analyzed spectral range, sample varieties, sample status, sample numbers, features (spectral features, image features, feature extraction methods), signal mode and data analysis strategies. The successful application of hyperspectral imaging in seed quality and safety inspection proves that many routine seed inspection tasks can be facilitated with hyperspectral imaging.

## Background

Nowadays, seed quality, which can be measured by its germinability or physicochemical attributes, has become increasingly important in the agriculture field due to the fact that it is a fundamental and critical factor in plant breeding and production. Seeds of high quality are a good start for plant growth, which indicates an abundant harvest. On the other hand, seed is often directly served as foodstuff, and its quality will therefore attract extensive attention. The seed quality is usually closely related to the eating quality, such as the texture, the flavour and the nutrient component. In order to meet the requirements of the consumers, seeds should be cautiously processed and stored after harvest. In the course of harvest, processing and storage, a fast, accurate and preferably non-destructive detection method of the seed quality is desired. Recently, hyperspectral imaging technique has been investigated as a potential analytical tool for non-destructive analysis and assessment of the seed quality and safety.

Hyperspectral imaging technology, which can acquire spectral and spatial information simultaneously, combines the advantages of spectroscopic and imaging techniques. In other words, it can simultaneously obtain the chemical information of heterogeneous samples and the spatial distribution of chemical components.

In recent years, hyperspectral technology has been widely used in the agriculture, food industry and medical industry etc. [1–4]. The potential or practical applications in seed industry include the detection of viability, vigour, defect, disease, cleanness and the seed composition determination. However, to our knowledge, a comprehensive literature survey on the seed quality and safety inspection using hyperspectral imaging has not been conducted, but should be widely desired. The motivation and purpose of this work is to summarize and analyse the development in seed quality and safety inspection by the hyperspectral technology.

## Application and conclusion of hyperspectral imaging for seed quality and safety inspection

### Seed variety classification and seed grading

#### Application

The varieties, grades, producing regions and storage conditions etc. all have influence on the nutrition and commercial price of seeds. What’s more, seed adulteration is also a great concern, which bothers planters and consumers, and sometimes can cause great losses. Thus, it endows the identification of seed variety and grade with great importance.

In general, there are two approaches for seed variety identification. The first one is the bulk samples detection, and the second one is the single seed identification. For seed variety identification based on bulk seeds, average spectra of each bulk are often extracted according to the predefined region of interest (ROI). For single seed identification, hyperspectral imaging can simultaneously acquire hyperspectral images of hundreds or thousands of single seeds. Spectrum of each single seed can be extracted, which makes it quite suitable for seed varieties classification to ensure the seed purity (Table 1).

**Table 1:** Summary of selected references applying hyperspectral imaging to seed classification and seed grading

Seed	Spectral range^a^	Varieties	Sample numbers	Features		Signal mode	Data analysis strategies		Main application type	Classification result (highest accuracy)	References
				Spectra/image	Extraction/selection methods		Analysis level	Classification/regression methods			
Barley, wheat and sorghum	1000–2498	1 variety of each kind of grain	150 of each kind of grain	Spectra	PCA	Reflectance	PW^b^ prediction map and OW^c^ (single kernels)	–	Grain topography classification	–	Manley et al. [19]
Black bean	390–1050 (501–1000)	3	300	Spectra and image	SPA, PCA, GLCM	Reflectance	OW (single kernels)	PLS-DA, SVM	Variety classification	98.33% (PLS-DA)	Sun et al. [15]
Grape seed	897–1752 (914–1715)	3 varieties, two growth soil	56	Spectra	PCA	Reflectance	OW (single kernels), PW PCA and prediction map	GDA	Assess Stage of maturation of grape seeds	> 95%	Rodríguez-Pulido et al. [22]
Grape seed	874–1734 (975–1646)	3	43,357	Spectra and image	PCA	Reflectance	OW (single kernels)	SVM	Variety classification	94.30%	Zhao et al. [26]
Maize	874–1734 (972–1642)	2 (transgenic and non-transgenic)	2100	Spectra	PCA, CARS	Reflectance	PW PCA and prediction map, OW (single kernels)	PLS-DA, SVM	Transgenic and non-transgenic classification	99.5% (PLS-DA)	Feng et al. [24]
Maize	400–1000	4 varieties, 3 crop years	3600	Spectra	no	Reflectance	OW (single kernels)	LS-SVM	Variety classification	91.50%	Guo et al. [12]
Maize	400–1000	4 varieties, 3 crop years	2000	Spectra	no	Reflectance	OW (single kernels)	LS-SVM	Variety classification	94.80%	He et al. [13]
Maize	400–1000	4 varieties, 3 crop years	2000	Spectra	no	Reflectance	OW (single kernels)	LS-SVM	Variety classification	94.40%	Huang et al. [11]
Maize	400–1000 (400–1000)	17	1632	Spectra and image	PCA, SPA, GLCM, MDS	Reflectance	OW (single kernels)	LS-SVM	Variety classification	94.40%	Huang et al. [17]
Maize	1000–2500	18	36	Spectra and image	PCA	Reflectance	OW (single kernels), PW PCA and prediction map	PLS-DA	Textural, vitreous, floury and the third type endosperm	85% (PLS-DA)	Manley et al. [20]
Maize	975–2570 (1101–2503)	3 hardness	115	Spectra and image	PCA	Reflectance	PW PCA and prediction map, OW (single kernels)	PLS-DA	Hardness classification	97% (PLS-DA)	Williams and Kucheryavskiy [18]
Maize	874–1734 (924–1657)	14	1120	Spectra	joint skewness-based wavelength selection	Reflectance	OW (single kernels)	LS-SVM	Variety classification	98.18%	Yang et al. [7]
Maize	874–1734 (975–1646)	3	12,900	Spectra and image	PCA	Reflectance	OW (single kernels)	SVM, RBFNN	Variety classification	93.85% (RBFNN)	Zhao et al. [25]
Maize	380–1030 (500–900)	6	330	Spectra and image	PCA, KPCA, GLCM	Reflectance	OW (bulk samples)	LS-SVM, BPNN, PCA, KPCs	Classes classification	98.89% (PCA-GLCM-LS-SVM)	Zhang et al. [78]
Rice	390–1050 (500–951)	4 origins	240	Spectra and image	PCA, GLCM	Reflectance	OW (single kernels)	SVM	Variety classification	91.67%	Sun et al. [16]
Rice	874–1734 (1039–1612)	4	225	Spectra	PLS-DA, PCA	Reflectance	PW PCA and OW (bulk samples)	KNN, PLS-DA, SIMCA, SVM, RF	Seed cultivars classification	100% (SIMCA, SVM, and RF)	Kong et al. [5]
Soybean, maize and rice	400–1000 (400–1000)	3 of each kind of seed	225 of each kind of seed	Spectra	neighborhood mutual information	Reflectance	OW (single kernels)	ELM, RF	Variety classification	100% (ELM)	Liu et al. [8]
Waxy corn	400–1000 (430–980)	4	600	Spectra and image	SPA, GLCM	Reflectance	OW (single kernels)	PLS-DA, SVM	Variety classification	98.2% (SVM)	Yang et al. [14]
Wheat	960–1700 (960–1700)	8	2400	Image	WT, STEPDISC, PCA	Reflectance	PW and OW (bulk samples)	BPNN, LDA, QDA	Classes classification	99.1% (LDA)	Choudhary et al. [79]
Wheat	960–1700 (960–1700)	8	2400	Spectra	STEPDISC	Reflectance	OW (bulk samples)	LDA, QDA, Standard BPNN, Wardnet BPNN	Variety classification	94–100% (LDA)	Mahesh et al. [6]
Wheat	960–1700 (960–1700)	5	2500	Spectra	STEPDISC	Reflectance	PW PCA and OW (bulk samples)	LDA, QDA	Classes classification	90–100% (LDA)	Mahesh et al. [9]

^a^The spectral range without brackets relates to the range acquisition of instrument, while the spectral range in brackets represents the spectral range for practical analysis

^b^PW means pixel-wise analysis, which is the analysis on the pixels

^c^OW means objective-wise analysis, which means the analysis on ROIs (ROI can be bulk, single kernel or self-defined)

Spectral features are the primary information utilized in the application of hyperspectral imaging system in the seed classification. Kong et al. used hyperspectral imaging to classify 4 varieties of rice seeds, and most of the discriminant models using full spectra and selected optimal wavelengths obtained good classification results (over 80%) [5]. Mahesh et al. used hyperspectral imaging to classify 8 wheat classes, and the results showed that the classification accuracy was over 90% for most of wheat classes [6]. Yang et al. used hyperspectral imaging to classify 14 maize varieties. Spectral information was extracted from each single seed, and discriminant models were established using full spectra and optimal wavelengths. The classification accuracy of most maize varieties was over 90% [7]. Liu et al. used hyperspectral imaging to classify the soybean, maize and rice. Spectral data were extracted and optimal wavelengths were then selected. Discriminant models using full spectra and optimal wavelengths all obtained good performances [8].

In addition to the classification of different varieties, studies of classification of different regions, years and attributes (such as moisture) of seeds are other research priorities. Mahesh et al. used hyperspectral imaging to identify wheat classes at five different moisture levels (12%, 14%, 16%, 18% and 20%). Wheat classes independent of moisture levels, moisture levels independent of wheat classes and wheat classes at different moisture levels were identified, respectively. Results indicated that classification of moisture levels were promising without considering wheat classes, while classification results of wheat classes with moisture levels considered were better than those without taking moisture level into consideration [9]. Then the same team used hyperspectral imaging to classify four wheat classes considering more variables. Particularly, seeds were collected with variations of crop year, growing location and moisture content. Their work showed that the NIR hyperspectral imaging could be used as a potential nondestructive tool for classifying moisture-specific wheat classes [10]. Huang et al. classified maize seeds of different years based on hyperspectral imaging. Classification models were developed using the least squares support vector machine (LS-SVM). To ensure the accurateness of the identification, incremental support vector data description was applied to update the LS-SVM model. The classification results of LS-SVM model combined with model updating was 10.3% higher than those of other non-updated models, demonstrating that the model updating could be an effective method for the identification of seeds of different years [11]. Guo et al. also proposed a model-updating algorithm for differentiating maize seed varieties from different years using hyperspectral imaging. The average classification accuracy was improved by 8.9%, 35.8% and 9.6% for the three test sets, respectively [12]. He et al. discriminated 4 varieties of maize seeds harvested in different years with the LS-SVM models updated with the clustering algorithm, which achieved an overall accuracy of 98.3% [13].

Except for spectral features, hyperspectral images also provide a large amount of image features. Choudhary et al. used hyperspectral imaging to classify eight wheat classes. The principal component (PC) score images were obtained by extracting the first three PC of each pixel after the pixel-wise principal component analysis (PCA) of hyperspectral images is performed. Based on the wavelet texture features, most discriminant models obtained decent results. Yang et al. extracted five morphological features (area, circularity, aspect ratio, roundness and solidity) and 8 texture features (energy, contrast, correlation, entropy and their standard deviations) from hyperspectral images to classify 4 waxy corn seed varieties. Support vector machines (SVM) and partial least squares discriminant analysis (PLS-DA) combining with spectral and appearance characteristic were employed to build classification models. The classification accuracy achieved by SVM models were more satisfactory than PLS-DA models [14]. Sun et al. extracted image features including four textural features (contrast, correlation, energy and homogeneity) and six morphological features (perimeter, area, major axis length, and minor axis length, eccentricity and equiv diameter) to classify black beans from 3 growing locations. In addition to SVM and PLS-DA methods, K-nearest neighbors was also used for model establishment. All the three methods were built based on spectral features, image features and the combination of spectral and image features, respectively [15]. From the studies of aforementioned two teams, they both extracted contrast, correlation and energy as textural features and area as morphological feature. These two studies both obtained good results with recognition accuracy more than 96% based on SVM model combining spectral and image features. Sun et al. used hyperspectral imaging to further study seed classification based on the combination of spectral features and image features. Both spectral features and image features were extracted from single rice kernels. Classification models were built using spectral features, morphological features, texture features, combinations of two kinds of features and the combination of all features, respectively. Results showed that models using all features performed better than other kinds of models for both full spectra and optimal wavelengths [16]. Huang et al. used hyperspectral imaging to classify 17 different varieties of maize kernels. Spectral features, image features were extracted. The dimension reduction method was applied to reduce the dimension of the combination of spectral features and image features. Discriminant models were built using only spectral features, combination of spectral features and image features and the dimension reduced data set of spectral features and image features, respectively. The overall results showed that models developed based on the combination of spectral features and image features and the reduced data set outperformed the model using only the spectral features [17].

The studies mentioned above mainly focused on the analysis of the single seed or quantity of seeds. They can be treated as object-wise (OW) analysis that uses average spectra of the depicted objects for data analysis. Apart from object-wise analysis, pixel-wise (PW) analysis is also an applicable method for seed quality determination [18–22]. For pixel-wise analysis, the spectra of individual pixels are used in the process of data analysis. Compared with object-wise analysis, pixel-wise analysis is more informative. Williams and Kucheryavskiy used hyperspectral imaging to classify maize kernels with three hardness categories (hard, medium and soft). Pixel-wise and object-wise PCA were used to indicate the differences between maize kernels with different hardness. Different methods (PLS-DA classification of individual pixels followed by a thresholding procedure, PLS-DA classification of kernels using mean spectrum of each kernel or score histograms of each kernel) were used to classify maize kernels. The classification results of models based on score histograms and mean spectra were significantly improved compared with models with a thresholding procedure [18]. Manley et al. used hyperspectral imaging to explore the influence of grain topography (sample shape and texture) using near infrared hyperspectral images. Kernels of three cereals (barley, wheat and sorghum) with varying topographic complexity were examined in the study. The influence of the topography on the spectral variation was examined using PCA and gradients classification. Classification gradients were defined according to score values, and color gradients corresponding to the classification gradients of score values were presented to assess the topographical effects within each PC. The results of classification gradient images and PC score plots showed that the prior PCs explained an accumulated total of 91.18%, 89.43% and 84.39% of the spectral variance, respectively, and all were influenced by kernel topography [19]. Manley et al. used hyperspectral imaging to detect endosperm texture in yellow maize. Hard, intermediate and soft maize kernels of three different genotypes were prepared. PCA was applied on hyperspectral images to form PCA scores images. PLS-DA models were built using PC scores, and the prediction maps were also formed [21]. Rodríguez-Pulido et al. used PCA to explore the differences of grape seeds of different varieties and in different growing soil. Important wavelengths were also selected to reduce the data volume and improve the speed of data analyses. Discriminant models based on full spectra or selected wavelengths both obtained good classification accuracy [22].

The aforementioned studies mainly focus on ordinary seeds. However, with the increasing concern about transgenic seeds all over the world, a fast and accurate detection method of transgenic seeds is also widely desired. Genotypic changes would bring about changes on molecular bonds such as C–H, C–N and C–O ultimately, thus it would be possible to evaluate the specific gene expression based on the phenotypic changes with the application of hyperspectral imaging [23]. Feng et al. used hyperspectral imaging to identify transgenic maize kernels. PCA was applied to hyperspectral images to explore the differences between transgenic and non-transgenic maize kernels. The visualization of classified maize kernels was also presented to show the pixel spectra combined with the spatial distribution of the maize kernel. Discriminant models were built using the full spectra or the optimal wavelengths. The overall results indicated that hyperspectral imaging could be used to identify transgenic and non-transgenic maize kernels [24].

Among all the factors, the sample volume plays an important role in the robustness of the results. The characteristics of hyperspectral imaging make it possible to acquire a large number of samples at the same time. Compared with other researches on seeds, sample preparation for seed variety and grading classification is simpler and more convenient. However, most of the current studies used small-volume samples, but a small sample volume couldn’t fully reflect the attributes of samples. Some studies have focused on the application of detecting large number of samples using hyperspectral imaging. Zhao et al. evaluated maize varieties with hyperspectral imaging and chemometrics methods. A total of 12,900 maize seeds of 3 different varieties were used in the experiment. Satisfactory results were obtained by the radial basis function neural network (RBFNN) model based on optimal wavelengths, with calibration accuracy being 93.85% and prediction accuracy being 91.00% [25]. Zhao et al. also discriminated grape seeds using hyperspectral imaging technique and multivariate analyses. Hyperspectral images were collected for 14,015, 14,300 and 15,042 grape seeds of three seed varieties. The results indicated that the variety of each single grape seed was accurately identified by SVM models based on effective wavelengths, with calibration accuracy being 94.3% and prediction accuracy being 88.7% [26]. The results of these two studies revealed that good results can be obtained with a large number of samples. In future studies, large number of samples are needed to establish universal, accurate and robust models.

#### Conclusion

For data acquisition, spectral features of object-wise spectra and pixel-wise spectra, image features and their combinations were all used in the seed variety and grading classification. Models using these features all obtained good results. However, spectral features are the most convenient and easy-to-obtain features, while the acquisition of image features are much more complex. Specially, more works on single kernels could be found, feature extraction of single kernels could represent the sample individuality. The abovementioned researches showed that the performance of models varied with different features but the differences were not significant. At the current stage, spectral features might be more suitable and applicable to develop real-world application compared with image features and the combination of image and spectral features.

The processing methods of hyperspectral images and the universality of classification models are main concerns for the application of hyperspectral imaging in the seed variety classification and the seed grading. It can be seen from Table 1 that some commonly used chemometrics methods, such as PLS-DA, artificial neural networks and LDA, have been applied to different seed researches and achieved good results. The universality of the model is the key to the practical application of hyperspectral imaging technology. In order to build database for seed variety classification and grading, a large number of samples are needed, which contains more characteristic information, such as water content, year, etc. [10–13]. On the other hand, in order to ensure the validity of models, it is also important to add appropriate upgrade methods to the conventional models. Huang et al., Guo et al. and He et al. all obtained good results with updated models [11–13].

In all, hyperspectral imaging has a very large practical prospect for the application in the seed variety classification and grading. In the future, researches on seed variety classification and grading should focus on the universality of models.

### Seed viability and vigor detection

#### Application

The seeds enter the aging process after natural maturity. During this process, the vitality of the seeds gradually decreases, which is a common phenomenon in the period of storage. Seed vigor is an important indicator synthesizing seed germination, seedling rate, seedling growth potential, plant stress resistance and production potential. For farmers, seeds with low viability will have low germination rate, which will increase the cost. Compared with seeds with low viability, seeds with high vigor have obvious growth advantages, which can save time, labor and material resources. Thus, an appropriate seed vigor detection method, such as hyperspectral imaging, can help farmers engage in agricultural production activities in a better way (Table 2).

**Table 2:** Summary of selected references applying hyperspectral imaging to seed viability and vigor detection

Seed	Spectral range^a^	Varieties	Sample numbers	Features		Signal mode	Data analysis strategies		Main application type	Classification result (highest accuracy)	References
				Spectra/image	Extraction/selection methods		Analysis level	Classification/regression methods			
Barley	900–1700 (1002–1626)	1 variety, 8 treatments	755	Spectra	PCA, MNF	Reflectance	PW^b^ prediction map and OW^c^ (single kernels)	Maximum likelihood multinomial, regression classifier	Germination level detection	97% when single kernels grouped into the three categories	Arngren et al. [28]
Corn	400–1000 (1000–2500)	3 varieties, 2 treatments	900	Spectra	No	Reflectance	OW (single kernels)	PLS-DA	Viability prediction	> 95.6%	Ambrose et al. [27]
Cryptomeria japonica and Chamaecyparis obtuse	400–980 (1250–2500)	2 treatments of each kind of seed	2320	Spectra	No	Reflectance	OW (single kernels)	Spectral index	Viability prediction	98.30%	Matsuda et al. [31]
Cucumber	400–1000(blue, green and red LED induced region)	1 variety, 2 treatments	200	Spectra	No	Reflectance	OW (single kernels), PW prediction map	PLS-DA	Viability prediction	100%	Mo et al. [32]
Muskmelon	948–2498	1 variety, 4 treatments	288	Spectra	VIP, SR, and SMC	Reflectance	OW (single kernels)	PLS-DA	Viability prediction	94.60%	Kandpal et al. [30]
Norway spruce	400–1000 (1000–2500)	1 variety, 3 treatments	1606	Spectra and image	L1-regularized logistic regression based feature selection	Reflectance	OW (single kernels)	SVM	Viability prediction	> 93%	Dumont et al. [29]
Pepper	400–1000(blue, green and red LED induced region)	1 variety, 2 treatments	600	Spectra	No	Reflectance	OW (single kernels), PW prediction map	PLS-DA	Germination level detection	> 85%	Mo et al. [33]
Tree seeds	392–889 (424–879)	3 varieties, 8 treatments	600	Spectra	LDA	Reflectance	OW (single kernels)	LDA	Germination level detection	> 79%	Nansen et al. [34]
Wheat, barley and sorghum	1000–2498	B: 3 varieties W: 3 varieties S: 2, varieties 6 treatments	1200	Spectra	PCA	Reflectance	OW (single kernels), PW prediction map	PLS-DA, PLSR	Viability prediction	R = 0.92 (PLS-DA)	McGoverin et al. [35]

^a^The spectral range without brackets relates to the range acquisition of instrument, while the spectral range in brackets represents the spectral range for practical analysis

^b^PW means pixel-wise analysis, which is the analysis on the pixels

^c^OW means objective-wise analysis, which means the analysis on ROIs (ROI can be bulk, single kernel or self-defined)

Ambrose et al. used hyperspectral imaging to evaluate the corn seed viability. Artificial aging was applied to obtain seeds with low viability, and germination test was conducted to determine seed viability as reference. Three different varieties of corn seeds (yellow, white and purple) were identified. Different spectra preprocessing methods and different spectral ranges (1000–2500 nm and 400–1000 nm) were explored. PLS-DA models were built to determine the viability of seeds. Visualization of treated and non-treated corn seeds were also achieved with hyperspectral imaging. The results demonstrated that the spectral range in the 1000–2500 nm performed better in the seed viability measurement [27]. Arngren et al. et al. used hyperspectral imaging to identify the pre-germinated barley. Eight pre-germination levels were prepared by setting pre-germination time of 0, 12, 18, 24, 30, 36, 48 and 60 h, and these levels were identified into three groups as normal, delayed and limited. PCA was conducted on the hyperspectral images to extract single kernel features. The maximum-likelihood multinomial regression classifier combined single kernel features were used to classify pre-germination degree of single barley kernels [28]. Dumont et al. used two hyperspectral imaging sensors, a thermal imaging system to identify viable seeds, empty seeds and seeds infested by *Megastigmus* sp. Larvae of Norway spruce (Picea abies), respectively. Images of single kernels were acquired and the spectral temperature features were extracted from these images. Results showed the feasibility of using hyperspectral imaging to identify viable seeds, empty seeds and seeds infested by *Megastigmus* sp. Larvae. Moreover, indices developed from the important wavelengths (1310 nm, 1710 nm and 1985 nm) showed good classification results, indicating the possibility to build an inexpensive devices [29]. Kandpal et al. used hyperspectral imaging to predict viability and vigor of muskmelon. Artificial aging was used to produce seeds with different levels of viability and vigor. Artificial aging periods were set a 0, 2, 4 and 6 days. Seeds were divided into three groups of vigor level after germination test, including the non-viable, 3-day germination (seeds germinated in 3 days) and 5-day germination. PLS-DA models were built to classify seeds at the three levels using full spectra and optimal wavelengths, respectively. The classification accuracy was over 88% [30]. Matsuda et al. used two hyperspectral imaging systems at different spectral ranges (400–980 nm and 1250–2500 nm) to identify sound and unsound Cryptomeria japonica (sugi) and Chamaecyparis obtuse (hinoki) seeds. There was a depression at 1730 nm corresponding to a lipid absorption band of sound seeds, and this depression could help to identify seed viability. A reflectance seed quality index (SQI) was proposed based on three identified wavelengths (1637 nm, 1734 nm and 1854 nm), which were selected according to the spectral depression of spectral reflectance curve. Such depression was obvious in sound seeds and absent or less prominent in unsound seeds. Average spectra based and pixel-wise spectra based SQI showed the feasibility to select sound seeds [31]. Mo et al. used a hyperspectral imaging system with various ranges of spectra induced by blue, green, red and RGB LED (400–500 nm for blue LED, 500–600 nm for green LED, 600–700 nm for red LED and 400–700 nm for RGB LED) to predict the germination quality of cucumber seeds. Artificial aging was used to produce aged seeds. PLS-DA was used to build classification models using spectra from blue, green, red and RGB LED illumination. The classification accuracy was over 90%. The results were verified by applying established models to the hyperspectral images to form prediction maps [32]. Mo et al. also used LED-induced hyperspectral imaging to detect viable and non-viable pepper seeds. Red, green and blue LEDs were used. Hyperspectral images were acquired under individual LED and three LEDs. Different spectral preprocessing methods were explored. PLS-DA models were used to build classification models, and classification accuracy was over 90%. Moreover, the germination test was conducted to evaluate the seed viability. PLS-DA models were used to form prediction maps [33]. Nansen et al. used hyperspectral imaging to evaluate the germination of seeds of *Acacia cowleana* Tate (Fabaceae), *Banksia prionotes* L.F. (Proteaceae), and *Corymbia calophylla* (Lindl.) K.D. Hill & L.A.S. Johnson (Myrtaceae) in Australia. Artificial aging was used to produce non-germinated seeds. LDA models were built to classify viable and non-viable seeds. The classification accuracy was over 78%, and differences existed in the classification results of three different tree species [34]. McGoverin et al. used the near-infrared hyperspectral imaging to classify viable and non-viable kernels of different cultivars of barley, wheat and sorghum. PLS-DA models were built to classify viable and non-viable kernels, and partial least squares regression (PLSR) models were used to predict the proportion of viable kernels in different incubation time of each kernel cultivar in hyperspectral images. Pre-germinated test by the tetrazolium test was used to determine the viability of kernels as reference. The results indicated that hyperspectral imaging could be used to identify viable and non-viable kernels of different kinds of crops with different cultivars [35].

The results in Table 2 verified the possibility of the seed viability and vigor detection using hyperspectral imaging, with all the accuracy higher than 90%. Most of the studies only used the spectral features. Different from the seed variety classification and seed grading, the seed viability and vigor detection needs to be verified with germination test, which increases the workload of researchers. Few samples were used in current experiments, but the volume of samples should be increased to establish a model for practical use. Specie differences of seeds are also key factors in seed viability and vigor detection. The different anti-aging ability of seeds should be taken into consideration in seed viability and vigor detection.

#### Conclusion

The current researches on seed viability and vigor detection mainly focused on healthy seeds with different anti-aging ability and unhealthy seeds (injury, insect pests, empty shells, etc.) with low viability. Compared with healthy seeds with different anti-aging ability, unhealthy seeds with low viability could be easily distinguished by obvious differences in hyperspectral imaging features. Artificial accelerated aging treatment is a commonly used method which is used to obtain seeds with different viability and vigor. However, there are still some differences between artificial accelerated aging treatment and naturally aging process. The acquisition of naturally aging seeds is one of the key difficulties in the promotion of using hyperspectral imaging in the practical researches of seed germination ability and vitality. Current researches prove the feasibility of using hyperspectral imaging in the detection of seed germination ability and vitality. In practical applications, the acquisition of naturally aged samples covers a large time span, and different naturally aging conditions also affect the characteristics of the samples. Thus, a universal database of seed viability and vigor detection using naturally aged samples is almost impossible to establish, so there is still a large distance to practical application. In future researches, cooperation between different research institutions is advised to help solve the problem of model sample sources and enrich the sample library.

### Seed damage detection

#### Application

During natural growth, transport and storage, seed damages caused by natural germination, insects, diseases and fungi might lead to the loss of yield and quality. Hyperspectral imaging can be used to identify and sort damaged seeds effectively (Table 3).

**Table 3:** Summary of selected references applying hyperspectral imaging to seed quality defect detection

Seed	Spectral range^a^	Varieties	Sample numbers	Features		Signal mode	Data analysis strategies		Main application type	Classification result (highest accuracy)	References
				Spectra/image	Extraction/selection methods		Analysis level	Classification/regression methods			
Mung bean	900–1700 (1000–1600)	1 variety, 8 treatments	2400	Spectra and image	PCA	Reflectance	OW^b^ (single kernels)	LDA, QDA	Insect damage detection	> 82%	Kaliramesh et al. [38]
Soybean	900–1700 with soft x-ray	1 variety, 5 treatments	1000	Spectra and image	GLCM	Reflectance	OW (single kernels)	LDA, QDA	Insect damage detection	99% (QDA)	Chelladurai et al. [39]
Wheat	700–1100	1 variety, 4 insect varieties	1500	Spectra and image	STEPDISC, GLCM, GLRM, PCA	Reflectance	OW (single kernels)	LDA, QDA	Insect damage detection	95.3–99.3%	Singh et al. [37]
Wheat	400–1000 (450–920)	1 variety, 3 treatments	144	Spectra and image	PCA	Reflectance	PW^c^ prediction map and OW (single kernels)	Spectral index	Seed sprouted detection	> 90%	Xing et al. [36]

^a^The spectral range without brackets relates to the range acquisition of instrument, while the spectral range in brackets represents the spectral range for practical analysis

^b^OW means objective-wise analysis, which means the analysis on ROIs (ROI can be bulk, single kernel or self-defined)

^c^PW means pixel-wise analysis, which is the analysis on the pixels

Natural germination of seeds during storage is one of the seed defects. Xing et al. used hyperspectral imaging system at the spectral range of 400–1000 nm to identify sprouted and severely sprouted wheat kernels. The sound kernels had a distinctly lower spectral reflectance in the wavelength region above 720 nm in contrast to sprouted kernels, while the reflectance of sprouted kernels peaked around 878 nm. Thus the ratio of reflectance at 878 and 728 nm were calculated as one of the indexes for seed defects discrimination. Score images of PC3 which could help to identify sprouted kernels more intuitively were also used as one of the indicators. Combined with the two indicators mentioned above, the classification accuracy of sprout damage in Canada Western Red Spring wheat was over 90% [36].

During the seed maturing and storage, the insect damage is another common damage in seeds. Preventing insect problems in the seeds is essential during the process of seed maturation and storage. Insects can feed on the seeds, multiply their population and spread viruses, which may cause serious consequences. Insects can cause pits on the surface or in the inside of the seeds, and insects may secrete harmful substances which could change the chemical composition of seeds. The spectral features and the image features will change accordingly, which makes hyperspectral imaging quite suitable for insect-damaged seed detection. Singh et al. used a hyperspectral imaging system and a color imaging system to identify healthy wheat kernels and midge-damaged wheat kernels from different locations in western Canadian. Discriminant models were built to classify healthy and midge-damaged wheat kernels. The overall average classification accuracy of most models was over 90% [37]. Kaliramesh et al. used a hyperspectral imaging system to classify the healthy mung bean and mung bean infested by Cowpea weevil (callosobruchus maculates F.). Spectral features and image features were extracted. Average classification accuracy more than 85% and 82% were obtained using statistical classifiers in identifying uninfected and infected mung bean kernels [38]. Chelladurai et al. used hyperspectral imaging to identify healthy soybeans and soybeans infested by Cowpea weevil (*Callosobruchus maculatus* (F.)). Degree of infestation was determined by form of insects in each soybean (egg, larvae, pupae and hollowed-out (with adults in the seed)). For hyperspectral images, significant wavelengths were identified by PCA loadings, and histogram features and spectral features of the selected significant wavelengths were extracted. Results showed that hollowed-out samples had the highest classification accuracy (98% and 99% for LDA and QDA, respectively) [39].

In recent years, the research on the seed safety inspection using hyperspectral imaging has been extended to the area of the fungus infection detection. Fungi is another severe damage to seeds. Under suitable environments, fungi could grow and spread quickly. Fungal growth would result in germination loss, discoloration, dry matter loss, increase in free fatty acids, heating, mustiness, and occasional production of mycotoxins [40]. Early and rapid detection of fungi infested seeds is important for the control of fungal growth and spread. Due to the characteristics of acquiring spatial and spectral information simultaneously, hyperspectral imaging has been widely used to detect seeds infested by fungi.

Wang et al. used hyperspectral imaging to detect Aflatoxin B1 on maize kernel surface. Different levels of Aflatoxin B1 were manually added to maize kernel surface. The classification accuracy of discriminant model using spectral features was 98% for different levels of Aflatoxin B1 [41]. Shahin and Symons used hyperspectral imaging to detect fusarium damaged Canada Western Red Spring wheat kernels. Healthy and damaged kernels with different damage degrees were studied. PCA analysis was conducted on the hyperspectral images, and standard deviation of scores of each kernel were extracted as features. Two modeling procedures of LDA were explored. Firstly, LDA model was used to classify sound and infected wheat kernels, and then LDA model was built to classify infected wheat kernels with different infection degree. The overall classification accuracy was over 80% for each category [42]. Senthilkumar et al. used hyperspectral imaging to detect barley infected by three fungi *Aspergillus glaucus*, *Penicillium* spp. and *Penicillium verrucosum*, respectively. Results indicated that after 4-week infection, the classification accuracy was 100% [43]. Senthilkumar et al. also used hyperspectral imaging to identify different stages of fungal infection (*Aspergillus glaucus* and *Penicillium* spp.) in canola. With the increase of fungal infection level, the classification accuracy increased from more than 90% at the initial infection stage to 100% [44]. Qiao et al. used hyperspectral imaging to detect fungi-contaminated peanuts of different varieties. Kernels fully besieged with fungi and healthy peanut kernels were acquired. Pixel-wise classification maps were obtained and the kernel-scale classification maps were also developed for qualitative analyses. The classification accuracy of calibration and validation sets was over 90% for different varieties of peanuts [45]. Lee et al. used hyperspectral imaging to detect watermelon seeds infected by *Acidovorax citrulli*. Classification accuracy of discriminant models was over 90%. Moreover, classification visualization images were obtained [46]. Lee et al. used hyperspectral imaging to detect watermelon seeds infected by cucumber green mottle mosaic viruses. Classification accuracy of discriminant models was over 83% [47]. Karuppiah et al. used hyperspectral imaging to detect fungal infection (Penicillium commune Thom, C. and A. flavus Link, J.) in five different pulses (chick peas, green peas, lentils, pinto beans and kidney beans). Two-way (healthy and beans with each infection level) and six-way (healthy and beans with different infection levels) classification models were built. All models obtained good performances, with classification accuracy over 80% [48]. Kandpal et al. used hyperspectral imaging to detect corn kernels contaminated by aflatoxin B_1_ (AFB_1_). Different varieties of corn contaminated by different concentrations of AFB_1_ were studied. Discriminant models were built to identify different contamination levels of corn kernels, and the classification accuracy was over 90% [49]. Jiang et al. used hyperspectral imaging to detect moldy peanuts. PCA analysis was conducted on hyperspectral images. A marker-controlled watershed algorithm was adopted to segment the kernels from the background. Threshold values were adopted to classify the infected pixels and infected kernels. The classification accuracy of learning and validation images was over 87% [50]. Del Fiore et al. used hyperspectral imaging to detect different varieties of maize kernels contaminated by different Aspergillus strains and different Fusarium strains. Optimal wavelengths were selected. Then analysis of variance (ANOVA) and significance of differences tested at the 95% confidence level by Fisher’s Least Significant Difference (LSD) test were conducted. The results showed that hyperspectral imaging is able to detect toxigenic fungi on maizes [51]. Barbedo et al. used a hyperspectral imaging system to detect wheat kernels contaminated by deoxynivalenol. Different varieties of wheat kernels produced in different years were used. A new index deoxynivalenol preliminary index (DPI) was proposed, and classification functions were developed based on DPI. The wheat kernels were divided into three classes and two classes based on different concentrations of deoxynivalenol. The classification functions obtained results with classification accuracy over 70% [52]. Zhu et al. used a fluorescence hyperspectral imaging to detect aflatoxins in corn kernels. Images were acquired for endosperm and germ side of maize kernels were acquired. The kernels were divided into different categories according to aflatoxins concentrations. Discriminant models were built using the two kinds of the spectral features. The classification accuracy was over 90% [53]. Yao et al. used the fluorescence hyperspectral imaging to detect single corn kernels infected with Aspergillus flavus. Narrow-band fluorescence indices were developed based on the extracted spectra, including the normalized difference fluorescence index (NDFI), the difference fluorescence index, and the ratio fluorescence index. Maximum likelihood and binary encoding classifiers were used to developed classification models [54]. Barbedo et al. used a hyperspectral imaging system to detect Fusarium head blight in wheat kernels. A Fusarium index (FI) was defined as the proportion of pixels with values over 0.58 in a kernel. Healthy and infected kernels were identified based on the threshold value of 0.5 of FI. The classification results were robust faced with factors such as shape, orientation, shadowing and clustering. The relationship between FI and deoxynivalenol were also explored, and good correlation indicated that hyperspectral imaging could be used to detect deoxynivalenol concentrations [55]. Yao et al. used a fluorescence hyperspectral imaging to detect maize inoculated with toxigenic (AF13) and atoxigenic (AF38) fungal strains. Hyperspectral images of germ side and endosperm side were acquired. Healthy kernels, infected kernels and kernels adjacent to the infected kernels were visually determined, respectively. Results of discriminant models indicated that the visually determined classes were not separated well. Specifically, classification accuracy of the maize kernels using 100 pb as threshold was over 90%. Results indicated that germ side was more effective for classification of contaminated and healthy maize kernels than endosperm side [56]. Serranti et al. used hyperspectral imaging to detect fusarium-damaged yellow berries and vitreous Italian durum wheat kernels. Bulk samples were used to build classification models, and images with single wheat kernels were used to validate the classification models. PCA was conducted for qualitative exploration of the separation of the three kind of wheat kernels. Discriminant models built using full spectra or optimal wavelengths all obtained decent results, with classification accuracy over 90% [57]. Wang et al. used hyperspectral imaging to detect aflatoxin B_1_ (AFB_1_) on maize kernel surface. Different concentrations of AFB_1_ were added to the kernels surface. PCA was conducted for the qualitative exploration of the separation of the different concentrations of AFB_1_. Discriminant models obtained good classification results, with minimum classification accuracy over 80% [58]. Williams et al. used hyperspectral imaging to detect fungal development (*F. verticillioides*) in maize kernels. Hyperspectral images were acquired under different stages of fungal development. PCA was conducted on hyperspectral images to explore the differences under different fungal growing time. Regression models were built to evaluate the fungal development degrees, with R^2^ of the calibration set over 0.8 and R^2^ of the prediction set over 0.7 [59]. Williams et al. used two hyperspectral imaging instruments to detect fungi infected maize kernels. PCA was firstly conducted on hyperspectral images to qualitatively identify healthy and infected maize regions. Discriminated models were applied on pixel-wise spectra to discriminate the infected and non-infected classes. R^2^ of two different hyperspectral imaging systems was both over 0.7 [60]. Tekle et al. used hyperspectral imaging to detect Fusarium-damaged oat kernels. Microscopy analysis was conducted for microanalysis of different Fusarium infection degrees. LDA model was applied to classify pixel-wise spectra within hyperspectral images to form classification maps of different degrees of infection. What’s more, based on the LDA model, ratio of damaged pixels in each grain was predicted [61]. Siripatrawan and Makino used hyperspectral imaging to monitor fungal growth on brown rice grains for 0, 2, 4, 6, 8 and 10 days. Scanning electron microscopy was also used to observe the fungal growth. Discriminant model was used for fungal growth degree determination, and then regression model for fungal colony counts was also developed, with R^2^ = 0.97, RMSEV = 0.39 1og(CFU/g) [62]. Chu et al. used hyperspectral imaging to detect AFB_1_ produced by *Aspergillus flavus* in single maize kernel of different varieties. Discriminant models were built to classify three different concentration levels of AFB_1_, with classification accuracy of calibration and prediction sets over 80%. Regression models were built to predict AFB_1_ content, with R^2^ of the calibration and prediction sets over 0.7 [63].

Although spectral features could reflect seeds suffering from fungal damage effectively, researchers have also used the image features to detect fungi infected samples. Singh et al. used hyperspectral imaging to detect wheat infected by Penicillium spp., Aspergillus glaucus and Aspergillus niger, respectively. PCA was applied to hyperspectral images, and significant wavelengths were identified by PCA loadings. After the PCA analysis, the mean, maximum and minimum reflectance intensities of the images at the significant wavelengths were calculated and used as inputs of discriminant models. Discriminant models obtained good results, with classification accuracy over 90%. However, there were difficulties in the detection of different fungal species [64].

As shown in Table 3, both the spectral features and image features were used to detect quality defects caused by pre-germination or insect damage. In these situations, the defects could be reflected by image information. As shown in Table 4, most of the studies of fungi damage on seeds used spectral features. The differences could be attributed to the defect types.

**Table 4:** Summary of selected references applying hyperspectral imaging to seed fungus damage detection

Seed	Spectral range^a^	Varieties	Sample numbers	Features		Signal mode	Data analysis strategies		Main application type	Classification result (highest accuracy)	References
				Spectra/image	Extraction/selection methods		Analysis level	Classification/regression methods			
Barley	900–1700 (1000–1600)	1 variety, 2 fungi	6300	Spectra and image	PCA	Reflectance	PW^b^ prediction map and OW^c^ (single kernels)	LDA, QDA, MDA	Fungus (*Ochratoxin A* and *Penicillium*) damage detection	> 82%	Senthilkumar et al. [43]
Canola	960–1700 (1000–1600)	1 variety, 2 fungi,	3300	Spectra and image	PCA	Reflectance	OW (single kernels)	LDA, QDA, MDA	Fungus (*Aspergillus glaucus* and *Penicillium* spp.) damage detection	> 90%	Senthilkumar et al. [44]
Corn	900–1700	3 varieties, 5 treatments	585	Spectra	No	Reflectance	OW (single kernels), PW prediction map	PLS-DA	Fungus (*Aflatoxin B1*) damage detection	96.90%	Kandpal et al. [49]
Corn	400–900 for fluorescence	1 variety, 3 treatments	492	Spectra	No	Reflectance	PW spectra	spectral index	Fungus (*Aflatoxin A. flavus*) damage detection	93%	Yao et al. [54]
Corn	400–701 for fluorescence, 461–877 for reflectance	1 variety, 3 treatments	300	Spectra	PCA	Reflectance	OW (single kernels), PW PCA	LS-SVM, KNN	Fungus (*Aflatoxin A. flavus*) damage detection	> 91% (KNN)	Zhu et al. [53]
Hick peas, green peas, lentils, pinto beans and kidney beans	960–1700 (1000–1600)	5 different pulses, 2 fungi	Over 10,000 kernels	Spectra and image	PCA	Reflectance	OW (single kernels), PW PCA	LDA, QDA	Fungus (*Penicillium commune Thom*, *C. and A. flavus Link*, *J.*) damage detection	96%-100%	Karuppiah et al. [48]
Maize	850–2800 (1000–2500)	4 varieties	120	Spectra	PCA	Reflectance	OW (single kernels), PW prediction map	SVM, SVR	Fungus (*Aflatoxin B1*) damage detection	R^2^ = 0.77	Chu et al. [63]
Maize	1000–2500	1 variety, 5 treatments	150	Spectra	PCA, FDA	Reflectance	OW (single kernels), PW PCA	FDA	Fungus (*Aflatoxin B1*) damage detection	88%	Wang et al. [58]
Maize	1000–2500	1 variety, 5 treatments	120	Spectra	PCA	Reflectance	OW (single kernels)	FDA	Fungus (*Aflatoxin B1*) damage detection	98%	Wang et al. [41]
Maize	960–1662 (1000–2498)	1 variety, 3 treatments	36	Spectra	No	Reflectance	OW (single kernels), PW prediction map	PLS-DA	Fungus (*Fusarium*) damage detection	77% (PLS-DA)	Williams et al. [60]
Maize	1000–2498	1 variety, nine treatments	160	Spectra	PCA, variable importance plots	Reflectance	OW (single kernels), PW PCA and prediction map	PLSR	Fungus damage detection	R^2^ = 0.87	Williams et al. [59]
maize	400–700	1 variety, 2 fungi, 3 treatments	180	Spectra	No	Reflectance	OW (single kernels)	discriminant analysis	Fungus (*Toxigenic and atoxigenic A. flavus*) damage detection	94.40%	Yao et al. [56]
Maize	400–1000	12 varieties, 4 fungi	Unknown	Spectra	PCA	Reflectance	OW (bulk samples), PW PCA	ANOVA, Fisher’s LSD test	Fungus (*Aspergillus strains*) damage detection	Fisher’s LSD test	Del Fiore et al. [51]
Oat50	1000–2500	1 variety, 4 treatments	180	Spectra	PLSR	Reflectance	OW (single kernels), PW prediction map	PLSR, PLS-LDA	Fungus (*Fusarium*) damage detection	R^2^ = 0.8	Tekle et al. [61]
Peanut	970–2570 (1000–2000)	1 variety, 2 treatments	149	Spectra	PCA	Reflectance	OW (single kernels), PW prediction map	PCA	Moldy kernel detection	98.73%	Jiang et al. [50]
Peanut	967–2499	1 variety, 2 treatments	More than 10,000 pixels	Spectra	ANOVA, NWFE	Reflectance	OW (single kernels), PW prediction map	SVM	Fungus (*Aflatoxin*) damage detection	> 94%	Qiao et al. [45]
Rice	400–1000	1 variety, 6 treatments	210	Spectra	No	Reflectance	OW (bulk samples)	SOM, PLSR	Fungus (*Aspergillus*) damage detection	R^2^ = 0.97	Siripatrawan and Makino [62]
Watermelon	948–2016	1 variety, 2 treatments	96	Spectra	Intermediate PLS (iPLS)	Reflectance	OW (single kernels) PW prediction map	PLS-DA, LS-SVM	Fungus (*Cucumber green mottle mosaic virus*) damage detection	83.3% (LS-SVM)	Lee et al. [47]
Watermelon	400–1000	1 variety, 2 treatments	336	Spectra	Intermediate PLS (iPLS)	Reflectance	OW (single kernels), PW prediction map	PLS-DA, LS-SVM	Fungus (*Acidovorax citrulli*) damage detection	> 90%	Lee et al. [46]
Wheat	528–1785	4 varieties, 2 fungi	803	Spectra	PCA	Reflectance	OW (single kernels), PW spectra	LDA	Fungus (*Fusarium*) damage detection	> 91%	Barbedo et al. [55]
Wheat	528–1785	33 varieties, 3 treatments	10,862	Spectra	No	Reflectance	OW (single kernels), PW spectra	spectral index	Fungus (*Fusarium head blight*) damage detection	81%	Barbedo et al. [52]
Wheat	400–1000 (450–950)	1 variety, 3 treatments	800	Spectra and image	PCA, STEPDISC	Reflectance	OW (single kernels)	LDA	Fungus (*Fusarium*) damage detection	92%	Shahin and Symons [42]
Wheat	900–1700 (1000–1600)	1 variety, 3 fungi	1200	Spectra and image	STEPDISC, GLCM, GLRM, PCA	Reflectance	OW (single kernels)	LDA, QDA, MDA	Fungus (*Penicillium* spp., *Aspergillus glaucus group*, *and Aspergillus niger*) damage detection	> 95%	Singh et al. [64]
Wheat	1000–1700 (1013–1650)	3 varieties	–	Spectra	PCA	Reflectance	OW (bulk, single kernels), PW PCA	PLS-DA, iPLS-DA	Fungus (*Fusarium*) damage detection	99%	Serranti et al. [57]

^a^The spectral range without brackets relates to the range acquisition of instrument, while the spectral range in brackets represents the spectral range for practical analysis

^b^PW means pixel-wise analysis, which is the analysis on the pixels

^c^OW means objective-wise analysis, which means the analysis on ROIs (ROI can be bulk, single kernel or self-defined)

From above researches, it can be seen that the researches on the seed damage can mainly be divided into the seed quality defect and seed fungal damage detection. For seed fungal damage, qualitative judgment and quantitative analysis were both performed in the references mentioned in Table 4. Good results have been achieved for seeds of different varieties, different fungal damages, most of which show accuracy above 90%.

#### Conclusion

Current researches indicate that hyperspectral imaging technique can detect whether seeds are affected by fungal damage, as well as the amount of toxicants produced by fungi to some extent. The abovementioned researches focused on several kinds of fungal damage and the produced toxicants. These studies showed the feasibility and repeatability of hyperspectral imaging to detect the seed fungal damage. More researches concerning more fungal damage types are needed. The main limit of the fungal damage detection lies in the detection limit of toxicant amount. At present, no research has yielded a specific result about the detection limits of early fungal infections that can be detected. For fungi such as *aflatoxin B1*, there are also uncertainties in the detection limit. The methods mentioned in the above references were not ideal for actual application, because the concentrations of fungi in these researches might exceed the concentrations in actual detection. The future studies can focus on the improvement of the detection limits. Besides, a large number of samples covering more sample features are also needed to establish a universal and robust model.

### Seed cleanness detection

#### Application

Seeds may be mixed with dry leaf or stalk pieces, or some other materials during harvest. Keeping seed clean is important for seed storage, trading and consumption. Studies that focused on examining spectral differences between different seeds and foreign materials (Table 5) are available in Table 5. Wallays et al. used a hyperspectral imaging system to detect material other than grain (MOG, such as chaff and straw) in different varieties of wheat, barley and corn. Spectral differences were observed between seeds and MOG. Genetic algorithm combined with PLS-DA was used to select sensitive wavelengths, and images at the selected wavelengths were used to detect the foreign materials, and prediction map was also formed [65]. Ravikanth et al. used a near-infrared hyperspectral imaging system covering the spectral range of 960–1700 nm to detect contaminants in Canada Western Red Spring wheat. Foreign materials (barley, canola, maize, flaxseed, oats, rye, and soybean), dockage types (broken wheat kernels, buckwheat, chaff, wheat spikelets, stones, and wild oats) and animal excreta types (deer and rabbit droppings) were studied. Spectral differences were observed between wheat and contaminants. Different spectral preprocessing methods and different discriminant models were used. Results of two-way classification models and multi-way classification models indicated the feasibility of using hyperspectral imaging to detect contaminants in wheat [66].

**Table 5:** Summary of selected references applying hyperspectral imaging to seed cleanness

Seed	Spectral range^a^	Varieties	Sample numbers	Features		Signal mode	Data analysis strategies		Main application type	Classification result (highest accuracy)	References
				Spectra/image	Extraction/selection methods		Analysis level	Classification/regression methods			
Wheat	960–1700 (1000–1600)	Extraneous materials (barley, canola, maize, flaxseed, oats, rye, and soybean), dockage types (broken wheat kernels, buckwheat, chaff, wheat spikelets, stones, and wild oats) and animal excreta types (deer and rabbit droppings)	4800	Spectra	No	Reflectance	OW^b^ (single particles)	SVM, NB and KNN	Foreign materials detection	> 80%	Ravikanth et al. [66]
Wheat, barley, corn	314–975 (403–950)	10 varieties, (material other than grain, such as chaff and straw)	More than 40,000 pixels	Spectra	GA-PLS-DA	Reflectance	PW^c^ spectra, PW prediction map	GA-PLS-DA	Foreign materials detection	–	Wallays et al. [65]

^a^The spectral range without brackets relates to the range acquisition of instrument, while the spectral range in brackets represents the spectral range for practical analysis

^b^OW means objective-wise analysis, which means the analysis on ROIs (ROI can be bulk, single kernel or self-defined)

^c^PW means pixel-wise analysis, which is the analysis on the pixels

#### Conclusion

As for seed cleanness detection, spectral features were the mostly used features. This phenomenon can be attributed to the spectral differences between the seeds and the foreign materials, and the spectral differences might be easier to be obtained rather than image features, especially for those foreign materials with great similarity. Previous studies (Table 1) have shown the possibility of seed variety classification. Same as the seed classification, seed cleanness detection is the classification of seeds and extraneous materials. Compared with the different varieties of seeds, significant differences could be found in spectral curves of extraneous materials such as straw, animal droppings, small stones, etc. The results in Table 5 indicate the feasibility of detecting different extraneous materials mixed with seeds using hyperspectral imaging. Future researches should improve sample size in order to enhance the robustness of models.

### Seed composition and properties determination

#### Application

Hyperspectral imaging has been widely used in seed compositions and properties determination. Moreover, the advantage of hyperspectral imaging makes it feasible to fast screen seed compositions and properties, especially for single seeds (Table 6). Zhang et al. used hyperspectral imaging to determine total iron-reactive phenolics, anthocyanins and tannins in wine grapes of skins and seeds of different varieties of wine grapes and different sampling dates. Outliers were identified and removed by a Monte–Carlo method. Different spectral preprocessing methods and different regression methods were used. As for grape seeds, the combination of MSC (used for spectral pretreatment) and SVR (used for model building) achieved the coefficient of determination (R^2^) over 0.8 for tannins and total iron-reactive phenolics [67]. Xing et al. used hyperspectral imaging to detect alpha-amylase activities in individual Canadian Western Red Spring (CWRS) wheat. A FT-Near-infrared spectrophotometer (FT-NIR) was also applied for comparison. PLSR model using spectral information from hyperspectral imaging performed better than FT-NIR, due to the fact that hyperspectral imaging also had the advantage of being able to localize the region where spectra were extracted from [68]. Wang et al. used hyperspectral imaging to predict textural properties (hardness, springiness and resilience) of maize kernels under different storage conditions. PLSR models were built using the full spectra or important wavelengths. Good prediction results were obtained with R^2^ of prediction over 0.7. The prediction maps of textural properties of single maize kernels were also obtained [69]. William et al. used two hyperspectral imaging systems to detect maize kernel hardness. Hard, intermediate and soft maize kernels were prepared. PCA was applied to hyperspectral images, and PCA scores image was formed to explore the classification of different hardness of maize kernels. PLS-DA models based on pixel-wise spectra obtained good performances with quite low root mean square error of prediction (RMSEP). Prediction maps were also obtained [70]. Sun et al. used hyperspectral imaging to detect the moisture content in the rice samples. Regression models were built using full spectra or optimal wavelengths. Both full spectra based models and optimal wavelengths based models obtained good results, and R^2^ of calibration and prediction sets of most models were over 0.9 [71]. Rodríguez-Pulido et al. used hyperspectral imaging to detect the flavanol in grape seeds. Grape seeds from two different varieties were collected, and flavanols determined by two different extract methods were studied. PCA analyses indicated that there were differences between two different varieties. PLSR models were built based on each variety and the combination of the two varieties. R^2^ of most models was over 0.8 [72]. Mahesh et al. used hyperspectral imaging to detect the protein content and hardness of Canadian wheat. Different varieties of wheat collected from different regions and different years were prepared. Hyperspectral images of bulk samples were acquired. Regression models were built using full spectra or optimal wavelengths, and class (variety) specific models and non-class specific models were built. The results showed that the variety had influence on prediction performances. Moreover, the correlation coefficient (r) was lower than 0.8 for protein and hardness of each variety [73]. Caporaso et al. used hyperspectral imaging to detect protein content in single wheat kernels. Wheat kernels were collected from 2013 to 2014, covering the wide variations caused by environment and agronomic conditions. Regression models were built to predict the protein content and kernel weight. Moreover, the impacts of different preprocessing methods on model performances were explored. Influences of kernel position, hardness and spectral region on model performances were also studied, and the first two factors showed little influence on the model performances. As for protein content prediction, R^2^ was over 0.7, but as for the kernel weight, R^2^ was much worse [74]. Cogdill et al. used hyperspectral imaging to detect the moisture and oil content in single maize kernels. Different spectral preprocessing methods and regression methods were studied. R^2^ for the moisture content prediction would reach 0.872, while R^2^ for the oil content was lower than 0.6 [75]. Weinstock et al. used hyperspectral imaging to detect oil and oleic acid concentrations in individual corn kernels. Different spectral ranges were used for determination of the oil and oleic acid concentrations, and the wavelengths were also selected by genetic algorithm. Images were acquired from germ side up or germ side down kernels. Moreover, hyperspectral imaging system was optimized by germ side, focal plane placement, orientation, temporal drift. Regression models obtained good performances, with R^2^ of most models over 0.6 [76]. Yang et al. used hyperspectral imaging to detect the protein content in wheat kernels. In total, eleven varieties of wheat were collected. Hyperspectral images of bulk wheat samples were acquired. Different spectral preprocessing methods and regression methods were explored. Good prediction results of the protein content were obtained with R^2^ of calibration and prediction over 0.9 [77].

**Table 6:** Summary of selected references applying hyperspectral imaging to seed composition

Seed	Spectral range^a^	Components	Sample numbers	Features		Signal mode	Data analysis strategies		Main application type	Classification result (highest accuracy)	References
				Spectra/image	Extraction/selection methods		Analysis level	Classification/regression methods			
Grape seed	865–1712 (977–1625)	Total iron-reactive phenolics, anthocyanins and tannins	60	Spectra	No	Reflectance	OW^b^ (single kernels)	PCR, PLSR, SVR	Regression	R^2^ = 0.91, 0.88, 0.90, respectively	Zhang et al. [67]
Grape seed	884–1717 (950–1650)	Flavanol content	99	Spectra	PCA, PLSR loadings	Reflectance	OW (bulk), PW^c^	PLSR	Regression	R^2^ = 0.88	Rodríguez-Pulido et al. [72]
Maize	700–1100 (750–1090)	Moisture and oil	473 for moisture, 151 for oil	Spectra	GA-PLS	Absorbance	OW (single kernels)	PLS	Regression	SECV = 1.20%, 1.38%, respectively	Cogdill et al. [75]
Maize	310–1100 (400–1000)	Hardness, springiness, and resilience	252	Spectra	SPA	Reflectance	OW (single), PW prediction map	PLSR	Regression	R^2^ = 0.90, 0.87, 0.85, respectively	Wang et al. [69]
Maize	950–1700	Oil and oleic acid	400	Spectra	GA-PLS	Reflectance	OW (single kernels), PW prediction map	PLS	Regression	RMSEP = 0.7%, 14%, respectively	Weinstock et al. [76]
Maize	960–1662 (1000–2498)	Hard, intermediate and soft	36	Spectra	PCA	Reflectance	OW (single kernels), PW PCA and prediction map, PLS-wise spectra model	PLS-DA	Classification	98%	William et al. [70]
Rice	871–1766	Moisture	120	Spectra	PCA, SPA	Reflectance	OW (bulk)	SVR, LS-SVR and bacterial colony chemotaxis LS-SVR (BCC-LS-SVR)	Regression	R^2^ = 0.98 (BCC-LS-SVR)	Sun et al. [71]
Wheat	980–2500 (1060–2500)	Protein	4150	Spectra	PLS	Reflectance	OW (single kernels), prediction map	PLS	Regression	R^2^ = 0.82	Caporaso et al. [74]
Wheat	1000–2500 (1235–2450)	Alpha-amylase activities	264	Spectra	No	Reflectance	OW (single kernels)	PLSR	Regression	R^2^ = 0.88	Xing et al. [68]
Wheat	860–1700	Hardness and protein	7200	Spectra	PLSR, PCR	Reflectance	OW (bulk)	PLSR, PCR	Regression	R^2^ = 0.88	Mahesh et al. [73]
Wheat	865–1711 (928–1695)	Protein	79	Spectra	No	Reflectance	OW (bulk)	PCR, PLSR, RBFNN	Regression	R^2^ = 0.92	Yang et al. [7]

^a^The spectral range without brackets relates to the range acquisition of instrument, while the spectral range in brackets represents the spectral range for practical analysis

^b^OW means objective-wise analysis, which means the analysis on ROIs (ROI can be bulk, single kernel or self-defined)

^c^PW means pixel-wise analysis, which is the analysis on the pixels

Spectral features were used in the detection of seed chemical compositions. The spectral features related to the chemical compositions according to the principles of the spectroscopy technique. The results of above references indicate that hyperspectral imaging technique can be used to detect the content of seed components, and the R^2^ of most researches can reach a satisfactory level. Another advantage of seed composition and properties determination using hyperspectral imaging is that the seed components can be visually distributed with PCA scores images, which make it possible to detect single seeds in industries.

#### Conclusion

From Table 6, good results have been achieved for various components detection of seeds. Current data sets are mainly based on a small amount of samples. Further researches should also focus on the sample size promotion. Apart from the component detection for a same seed variety, the same component in different seed varieties also should be taken into consideration in order to improve the universality of models. Same as the seed fungi damage detection, detection thresholds of low-content components should also be paid attention to.

## Summary of data analysis

As a fast and non-destructive method, hyperspectral imaging has been widely applied in the seed quality and safety inspection. In this review, the applications of this technique involve the seed classification and grading, viability and vigor detection, damage (defect and fungus) detection, cleanness detection and composition determination. The summary for each category is presented in Tables 1, 2, 3, 4, 5 and 6. These Tables are further summarized in this section, including the analyzed spectral range, signal mode, sample numbers, features (spectral features, image features and feature extraction methods), spectral preprocessing methods and data analysis strategies.

Researches have showed that different spectral wavebands can be adopted for the detection with a same purpose, and satisfactory results could be achieved. Thus, researchers can select the wavebands depending on their practical conditions. For example, the 972–1642 nm and 400–1000 nm wavebands were utilized by Feng et al. [24] and He et al. [13] respectively, and they both accomplished classification accuracy over 90% in the maize variety classification.

Although there are three different signal modes for hyperspectral imaging (i.e. reflectance, transmittance and interactance), all the references in the Tables adopted the reflectance mode. The reason might be that the reflectance mode could detect internal quality features as well as external quality features, such as shape, size and surface texture, and that the reflectance mode is simple and easy to operate. As discussed above, the requirement of equipment with certain spectral wavebands and modes is not compulsory, and the selection of certain spectral wavebands and modes mainly depend on the researchers.

When it comes to the extraction of spectral features, PCA [16, 19, 28, 50, 64, 78] is the most common method. PCA can transform a set of variables with possible correlations into a set of linearly independent variables by the orthogonal transformation. The first few principal components contain most of the information. Therefore, PCA can not only be utilized in the qualitative analysis of spectral data (e.g. PCA score plot or PCA score image visualization), but also help to select the characteristic wavelengths according to the PCA loadings for the quantitative analyses. Hyperspectral imaging will generate a large amount of data. Extracting useful features from the large amount of data can significantly reduce the data volume, and therefore increase the computation efficiency. In addition to PCA, successive projections algorithm (SPA) and stepwise discriminant analysis (STEPDISC) are also commonly used methods in characteristic wavelengths selection. In this review, characteristic wavelengths selection by SPA was applied in the seed classification, grading and composition determination [14, 15, 71]. STEPDISC was utilized in not only the seed classification and grading but also the seed damage detection [6, 9, 36, 42, 79].

Apart from spectral features, image features, such as texture features, color features (HSV and RGB), morphological features (perimeter, area and roundness, etc.), and statistical features of gray value also showed great potential in the seed quality and safety detection [14, 17, 20, 29, 78, 79]. Gray level co-occurrence matrix (GLCM) can reflect the comprehensive information of image grayscale about directions, adjacent intervals and amplitudes of variations, which makes it the most commonly used image feature selection method [14, 15, 37, 39, 78]. However, spectral features is still the mostly used information in hyperspectral imaging data analysis, which may be due to its convenience of acquisition. Models based on the combination of spectra and image usually obtained superior results compared with models using only spectral features or image features. The results of models using only image features are usually inferior to those of models based on spectral features [14–16, 38]. The analysis of spectral features is easier than that of image features, and results have proved the efficiency of models based on spectral features. Given this background, most of the references focus on only the spectral features [12, 13, 22, 30, 31, 33, 57, 61, 67].

After the acquisition of spectra, preprocessing methods were adopted by some researches to denoise the spectra and therefore improve the performance of the model. Normalization, standard normal variate (SNV), multiplicative scatter correction (MSC) and savitzky–golay (1st and 2nd derivative) smoothing are commonly used spectra preprocessing methods. Normalization is used to normalize data and fit the data within 0–1, which can reduce the spectral difference caused by the inconsistent height of the sample surface. SNV is often applied in scatter correction to attenuate the slope variation of spectra. MSC is the most commonly method which could be used to remove the undesirable scatter effect. Savitzky–golay smoothing can be used to eliminate spectral noise, such as baseline-offset, tilt and reverse, etc. Ambrose et al. [27] used all these pretreatment methods in their research. As for the vigor detection of different varieties of corns, the optimal preprocessing method varied. In the vigor detection by the visible near infrared, the 2nd derivative savitzky–golay smoothing performed best for the yellow corn while the 1st derivative savitzky–golay smoothing was more suitable for the white corn and the purple corn. For the same sample, the best preprocessing method may be different for different spectral bands. In the vigor detection of purple corn by the short-wave near-infrared, the accuracy could be improved by the MSC and SNV spectral preprocessing. Therefore, there is no definite selection criteria for the spectral preprocessing method and it needs to be selected according to the practical application situation.

Calibration models are of significant importance in seed quality and safety inspection. For discriminant models and regression models, PLS was the widely used chemometric method for data analyses of hyperspectral images. PLS had the characteristic of dealing with large number of data rapidly and efficiently, and it worked well for both discriminant (PLS-DA) [14, 27, 30, 32] and regression analysis (PLSR) [31, 68, 72]. Except for PLS, neural network (BPNN), LDA, QDA, SVM, LS-SVM, PCA were also widely used chemometric methods. As supervised linear discriminant analysis models, PLS-DA, LDA and QDA have a wide range of applications in early studies using hyperspectral imaging techniques to detect the seed quality and safety [6, 14, 35, 42, 46, 57, 79]. Although BPNN, SVM and LS-SVM are also supervised discriminant analysis models, they have excellent performance in nonlinearly separable problems, so these methods are often used to build models [5, 6, 9, 47, 79]. In addition to the commonly used modeling approaches mentioned above, RF, KNN, SIMCA, FDA, GDA, etc. have also been used to establish models [5, 8, 22, 41]. Performances of discriminant and regression models varied due to their different principles, so the modelling approach should be selected based on the actual situation. For example, Feng et al. [24] and Yang et al. [14] both utilized PLS-DA and SVM in the classification of maize. PLS-DA achieved an accuracy of 99.5% in the former study while SVM performed best in the latter with the accuracy being 98.2%.

In sum, the use of these chemometric methods showed their effectiveness in hyperspectral image analysis. Researchers conducted data analysis procedures based on their own demands and interests, which resulted in the use of many different methods. Indeed, the optimal methods for data analysis could not be simply identified. Most of the studies used small samples volume, so the universality and robustness of these methods needed to be verified using large amount of samples in further studies to meet the demand of practical real-world application.

## Opportunities and challenges

Hyperspectral imaging, as mentioned above, has the advantage of acquiring the spectral features and spatial features simultaneously. This advantage makes it quite convenient for researchers to define the study region within hyperspectral images. Seed is quite suitable for hyperspectral image analysis, in the forms of single seeds or bulk samples. Researches show a great potential of applying hyperspectral imaging to seed quality and safety inspection.

As for single seeds, there would be hundreds of seeds in one hyperspectral image, which could highly increase the sampling and detection efficiency. With the high efficiency of sampling, more seeds with more variations could be used for analyses, and hyperspectral image databases of seeds could thus be established and studied. The high sampling efficiency endows hyperspectral imaging with great potential in real-world industrial application.

Discriminant and regression models were built using limited number of samples, and the number of samples could increase to a certain number due to the high sampling efficiency. With more samples covering more variations, representative discriminant models could be built for real-world industrial application. In recent years, the deep learning has been used in various fields as an effective modelling tool. Deep learning has the obvious advantage of dealing with large amount of data, which can learn and extract sample features automatically. Qiu et al. used hyperspectral imaging combined with the deep learning to identify rice seed varieties and achieved good results. Deep learning has great potential of using hyperspectral imaging in seed quality and safety inspection [80].

Hyperspectral imaging also provides the feasibility to obtain visualization prediction maps, which could be helpful for industrial applications. With the development of hardware and software, the computation time and efficiency has been significantly improved. The large amount of data generated by hyperspectral images can be dealt with in a high efficient way. Still, how to build models with such a large number of samples remains as a challenging problem.

Although great opportunities could be foreseen, great challenges are still on the road. A quite common challenge is the development and maintenance of calibration models. Universal and representative calibration models are the basis of real-world application of hyperspectral imaging. But it is quite difficult to build such calibration models, due to the fact that great variations caused by varieties, growth condition, growth location, crop years etc. exist. Although high sampling efficiency can help to cover more variations, which makes model maintenance more efficient. Besides, model maintenance is still a complex issue. Moreover, as can be seen in Tables 1, 2, 3, 4 and 5, various data analysis methods and strategies have been used for hyperspectral image analysis. However, one or few optimal strategies of data analysis should be selected for real-world application.

## Conclusion

Hyperspectral imaging is a complex, highly multidisciplinary field with the aim of realizing efficient and reliable measurement of both contents and spatial distributions of multiple chemical constituents and physical attributes simultaneously without monotonous sample preparation, and therefore offering the possibility of designing inspection systems for the automatic grading and defects determination of seeds. The various applications outlined in this review show the capability of using hyperspectral imaging for seed grading, viability and vigor detection, defect and disease detection, cleanness detection, and seed composition determination. Moreover, some practical implementations for real-time monitoring are currently available. It can be anticipated that real-time seed monitoring systems with this technique will meet the requirements of the modern industrial control and sorting systems of seeds in the near future.

## Data Availability

Not applicable.

## Abbreviations

ANOVA: analysis of variance
BPNN: back propagation neural network
CARS: adaptive reweighted sampling method
DPI: preliminary index
ELM: extreme learning machine
FDA: factorial discriminant analysis
FI: fusarium index
GA-PLS: genetic algorithm partial least squares
GA-PLS-DA: genetic algorithm partial least squares discriminant analysis
GDA: general discriminant analysis
GLCM: gray level co-occurrence matrix
GLRM: grey level run length matrix
KNN: K-nearest neighbor algorithm
KPCA: Kernel principal component analysis
KPCs: Kernel principal components
LDA: linear discriminant analysis
LS-SVM: least squares-support vector machine
LS-SVR: least squares-support vector regression
MDA: multiple data analysis
MDS: multidimensional scaling
MNF: minimum noise fraction
MOG: material other than grain
MSC: multiplicative scatter correction
NB: Naïve Bayes
NDFI: normalized difference fluorescence index
NDVI: normalized difference vegetation index
NWFE: nonparametric weighted feature extraction
OW: object-wise
PC: principal component
PCA: principal component analysis
PLS-DA: partial least squares discriminant analysis
PLSR: partial least squares regression
PW: pixel-wise
QDA: quadratic discriminant analysis
RBFNN: radial basis function neural network
RF: random forest
RMSEP: root mean square error of prediction
SECV: standard error of crossvalidation
SIMCA: soft independent modeling of class analogy
SMC: significance multivariate correlation
SNV: standard normal variate
SOM: self-organizing map
SPA: successive projections algorithm
SQI: seed quality index
SR: selectivity ratio
STEPDISC: stepwise discriminant analysis
SVM: support vector machine
SVR: support vector regression
VIP: variable importance in projection
WT: wavelet transform
